# Primiparous and Multiparous Sows Have Largely Similar Colostrum and Milk Composition Profiles Throughout Lactation

**DOI:** 10.3390/ani9020035

**Published:** 2019-01-26

**Authors:** Jessica R. Craig, Frank R. Dunshea, Jeremy J. Cottrell, Udani A. Wijesiriwardana, John R. Pluske

**Affiliations:** 1 Research and Innovation, Rivalea (Australia) Pty. Ltd., Corowa, NSW 2646, Australia; 2 School of Veterinary and Life Sciences, Murdoch University, Murdoch, WA 6150, Australia; j.pluske@april.org.au; 3 Faculty of Veterinary and Agricultural Sciences, The University of Melbourne, Parkville Vic 3010, Australia; fdunshea@unimelb.edu.au (F.R.D.); jcottrell@unimelb.edu.au (J.J.C.); udani.wijesiriwardana@unimelb.edu.au (U.A.W.); 4 Australasian Pork Research Institute Ltd. (APRIL), Willaston, SA 5118, Australia

**Keywords:** colostrum, energy, fat, immunity, IgG, lactose, milk, parity, piglet, protein

## Abstract

**Simple summary:**

Progeny born to primiparous sows (gilt progeny) are born lighter, grow slower, and have higher rates of mortality than piglets born to multiparous sows. An understanding of why this might be the case is necessary to implement management strategies that improve the health and welfare of first-litter piglets and increase their production efficiency. Differences in the composition of colostrum and milk between primiparous and multiparous sows might be a contributing factor. Results from this study suggest that primiparous and multiparous sows (parities 3 and 4) have similar levels of immunoglobulin G and energetic components (net energy, fat, protein, and lactose) in colostrum and milk throughout the course of lactation. However, lower lactose levels from day 14 onwards in milk from primiparous sows compared to milk from multiparous sows may indicate that there is a reduction of lactose uptake into the mammary gland in primiparous sows during late lactation, which may reduce milk volume. Lower growth rates and higher rates of mortality in gilt progeny are therefore likely to be as a result of lower colostrum and milk production in primiparous sows and (or) an inability of their piglets to digest these nutritional components effectively.

**Abstract:**

It is important to understand the biological factors influencing the poorer lifetime performance of gilt progeny in comparison to sow progeny and determine whether this may be partially due to differences in lactation performance between primiparous and multiparous sows. It was hypothesized that primiparous sows would have lower levels of immunoglobulin G (IgG) in colostrum and milk compared to multiparous sows, and lower levels of other energetic components. Differences in colostrum and milk composition between ten primiparous and ten multiparous sows (parities 3 and 4) from a commercial herd were examined throughout lactation (day 0, 1, 2, 3, 7, 14, and 21). Overall, there were no (*p* ≥ 0.05) parity differences in total IgG, fat, protein, lactose, and net energy (NE) concentrations. Primiparous sows had higher lactose levels at day 2 (parity by timepoint interaction; *p* = 0.036) and lower NE at day 3 (*p* = 0.091), and multiparous sows had higher lactose levels at days 14 and 21. Results suggest that shortcomings of gilt progeny are unlikely due to insufficient nutrient levels in colostrum and milk, and more likely to reduced colostrum and milk intake and their capacity to digest and absorb each component.

## 1. Introduction

Progeny born to primiparous sows (gilt progeny) are born lighter, grow slower, and have higher rates of mortality than progeny born to multiparous sows [1,2,3]. As this results in high costs to the producer, an understanding of why these shortcomings occur is required to successfully implement management strategies to improve the health and welfare of gilt progeny. A possible explanation for this divergence is different quality and (or) quantity of colostrum and milk in primiparous compared to multiparous sows. Colostrum yield is difficult to measure [4,5] and therefore differences between primiparous and multiparous sows are relatively unknown. It is speculated that multiparous sows produce more milk once lactation is established [6] due to having a larger udder [7] and being able to convert more energy into milk production compared to the primiparous sow, who is still partitioning energy into her own maturation and growth [8]. There is an inference that the colostrum and milk of primiparous sows is lower in immunoglobulins (Igs) and other components (total energy, fat, protein, and sugars) than in multiparous sows due to the naïve nature of her immature immune system [9], lower gastric capacity and resulting lower feed intakes [5,10]. However, data to support this notion are equivocal and differ greatly in terms of their experimental design, with genetics [11,12,13], season [13], management factors such as vaccination [14] and medical treatment [15], nutrition [4,16], parity numbers involved [17], timing of colostrum and milk sampling [5], udder section [4,18,19,20], oxytocin use [21], and farrowing induction [22,23] all affecting the composition of lacteal secretions. 

This study investigated differences in total concentrations of immunoglobulin G (IgG), macronutrient levels (protein, fat, and lactose), and net energy (NE) content in colostrum and milk between primiparous and multiparous sows (parities 2 and 3) from farrowing to day 21 of lactation. It was hypothesized that primiparous sows would have lower concentrations of IgG in colostrum and milk compared to multiparous sows, coupled with lower levels of macronutrients, resulting in lower overall NE content of their lacteal secretions compared to that of older sows.

## 2. Materials and Methods 

### 2.1. Ethics Statement

All experimental procedures carried out were approved in May 2016 by both the Rivalea (Australia) Animal Care and Ethics Committee (protocol number 16P030) and the Murdoch University Animal Ethics Committee (protocol number N2847/16) in accordance with the Australian Code for the Care and Use of Animals for Scientific Purposes [24].

### 2.2. Experimental Animals

The experiment was conducted under commercial conditions at a large piggery in New South Wales, Australia (Rivalea Australia Pty Ltd; Corowa NSW, Australia), from May to July 2016. Ten F1 primiparous sows (parity 1) and 10 F1 multiparous sows (parities 3 and 4 at the current lactation; PrimeGro^TM^, Corowa NSW, Australia) were used. Sows were allocated to the study based on expected farrowing date, parity, and success in lactation. All sows were fed common gestation (12.7–13.2 MJ DE/kg, 12.1–13.1% CP, 0.5% available Lys, as-fed basis) and lactation (14.81 MJ DE/kg, 16.1–16.8% CP, 0.8–0.9% available Lys) diets throughout the experiment. Sows were housed in groups of 40 during gestation (approximately 2 m^2^ per animal), with primiparous sows housed separately to multiparous sows. Sows entered the farrowing room at approximately day 110 of gestation and were housed in farrowing crates fitted with drinker nipples for the dam and piglets, and a heat lamp in the creep area. Dams were spread over four separate farrowing rooms according to expected due date and farrowed over a period of 15 days. Piglets were fostered at least 24 h after farrowing according to commercial practices to standardize litters to match the number of productive teats and minimize within-litter weight variation. During lactation, sows were fed according to the commercial feeding regimen and given an allowance of 2.5 kg/d of lactation feed from entry to farrowing, 2.5 kg on day 0, 3 kg on day 1, up to 4 kg on day 2 and ad libitum access thereafter until weaning. Piglets were not given access to creep feed and were weaned at 26.5 (± 0.4) days of age.

### 2.3. Colostrum and Milk Collection

A colostrum sample (approx. 5 mL) was collected from each sow at farrowing (day 0; within 1 h from birth of the first piglet), day 1 (approx. 24 h later) and day 2 (approx. 48 h) after farrowing without the use of oxytocin. Milk samples were collected on days 3 (approx. 72 h), 7, 14 and 21 after farrowing following a 1 mL subcutaneous (intravulval) injection of oxytocin (10 IU; Ilium Syntocin, Troy Laboratories, Glendenning NSW, Australia). Colostrum and milk samples were pooled from as many teats as possible, collected throughout the letdown phase, and immediately frozen at −20 °C until further analysis. Several colostrum and milk samples were unable to be collected, or an insufficient amount was obtained for each subsequent laboratory analysis and therefore not all dams were represented at each timepoint in the results for all colostrum and milk composition parameters. Numbers of observations (*n*) per parameter by dam parity (primiparous or multiparous) for colostrum and milk composition traits are given in Table 1.

### 2.4. Assay for Total Fat

Whole colostrum and milk samples were assayed for total fat using a method adapted from the UV visible photometric method by Forcato et al. [25]. A standard colostrum sample was used for each assay, obtained from a pooled sample of colostrum collected from several unrelated commercial sows during farrowing. The standard sample was frozen in 1 to 2 mL aliquots at −20 °C, one of which was sent to an external laboratory (NSW DPI Laboratory Services, Wagga Wagga NSW, Australia) and tested for total crude fat via Soxhlet extraction. The crude fat level of this sample was evaluated as 5.6%, and this sample was diluted using ultrapure water to 2.8%, 1.4%, 0.7% and 0.35% for the standard curve for each assay, with ultrapure water used as a blank. 

Sixty μL of colostrum, milk, or standard was added to 3 mL of chilled, pure ethanol (99.5% min.; Ajax Finechem; Thermo Fisher Scientific, Scoresby Vic, Australia) in a sterile glass tube and briefly mixed. The suspension was then frozen at −20 °C for 1 h and subsequently transferred to small polystyrene tubes and spun at 13,000 × g for 15 min at 4 °C. The supernatant was then transferred to another sterile glass tube and left to reach room temperature. From this solution, 200 μL of each standard and sample was added to a 96 well UV compatible assay plate (UV-Star Microplate, Grenier Bio-One; Interpath Services Pty. Ltd., Heidelberg West Vic, Australia) in duplicate and read at 208 nm on a plate reader (Tecan Spark; Tecan Group Ltd. Männedorf, Switzerland). A standard curve was generated from the absorbance readings for each standard dilution using online software [26] and total fat values for each sample were calculated using this curve. The assay had an intra-assay CV of 1.1% and an inter-assay CV of 8.5%.

### 2.5. Total IgG, Protein, Lactose, and Energy Calculation

Colostrum and milk samples were spun at 21,000 × g for 40 min at 4 °C to remove fat prior to analysis for IgG, total protein, and total lactose. Concentration of IgG was determined using a commercial pig IgG ELISA kit (Bethyl Laboratories, Montgomery TX, USA; 5.5% intra-assay CV and 29.1% inter-assay CV). Total protein was determined using the Pierce BCA Protein Assay Kit (Thermo Fisher Scientific, Scoresby Vic, Australia) after dilution of standards and samples in 2% sodium dodecyl sulfate (SDS; Invitrogen Life Technologies; Thermo Fisher Scientific, Scoresby Vic, Australia) to remove any interference from remaining lipids as per the procedures used by Geale [11]. The intra- and inter-assay CV for the protein assay were 1.9% and 4.5%, respectively. Total lactose was determined using a commercial colorimetric assay (BioVision; Sapphire Bioscience, Redfern NSW, Australia; 3.2% intra-assay CV and 6.0% inter-assay CV). Total NE (on an as-fed basis) of milk from day 3 of lactation onwards was calculated from the values for total fat, protein, and lactose according to the equation derived by Hansen et al. [27];

NE (MJ/kg) = 0.389 × Fat (%) + 0.239 × Protein (%) + 0.165 × Lactose (%).

Total NE of colostrum on days 0, 1, and 2 were calculated as adapted by Theil et al. [28] using the same equation, without values for protein since approximately 90% of protein at this stage is represented by immunoglobulins that are not used as an energy source for the neonatal piglet [28,29].

### 2.6. Statistical Analysis

Farrowing data and colostrum and milk IgG concentration at each individual timepoint (day 0, 1, 2, 3, 7, 14 and 21) were analyzed as linear mixed models with dam parity as a fixed factor. Colostrum and milk macronutrient composition data were analyzed as a repeated measures mixed model with dam parity, timepoint, and their interaction as fixed factors, with timepoint defined as a repeated measure with a heterogeneous autoregressive first order covariance structure. Parameter estimates were calculated using the method of restricted maximum likelihood (REML). All data analysis was carried out using the MIXED procedure of SPSS (IBM SPSS, version 24; IBM, Armonk NY, USA) with dam as the experimental unit. 

Any extreme outliers were carefully considered for accuracy and either removed or kept in the analysis as appropriate (reflected in the number of observations for each trait, given in Table 1). Farrowing room was tested as a random effect and gestation length, days from entry to the farrowing room until farrowing, and born alive were tested as covariates for each trait and were kept in or left out of the model as appropriate. As such, none of these factors has a significant effect on the overall model for any traits (*p* ≥ 0.10) and the final model for each trait was therefore the simple factorial model. Pairwise comparisons were made between individual treatment means using the least significant difference (LSD) method, and comparisons between interaction means were made when the interaction was significant (*p* < 0.05) or a trend (*p* < 0.10) by simple effects analysis in SPSS syntax using the COMPARE function. Estimates stated herein are reported as mean ± standard error of the mean (SEM).

## 3. Results

### 3.1. Farrowing Performance

One primiparous sow was removed from the experiment after the day 7 milk sample as her piglets were unthrifty and her udder was drying up; samples from this animal were excluded from the analysis. Primiparous and multiparous sows had similar gestation lengths (114.9 ± 0.5 vs. 115.8 ± 0.5 days, respectively; *p* = 0.18), number of stillbirths (0.3 ± 0.2 vs. 0.7 ± 0.2, respectively; *p* = 0.26), mummified fetuses (0.4 ± 0.2 vs. 0.1 ± 0.2, respectively; *p* = 0.32) and number of piglets weaned (9.6 ± 0.5 vs. 10.6 ± 0.5, respectively; *p* = 0.18). Primiparous sows had less piglets born alive (*p* = 0.031) than multiparous sows (10.7 ± 0.9 vs. 13.7 ± 0.9, respectively).

### 3.2. Immunoglobulin G

**Figure 1:**
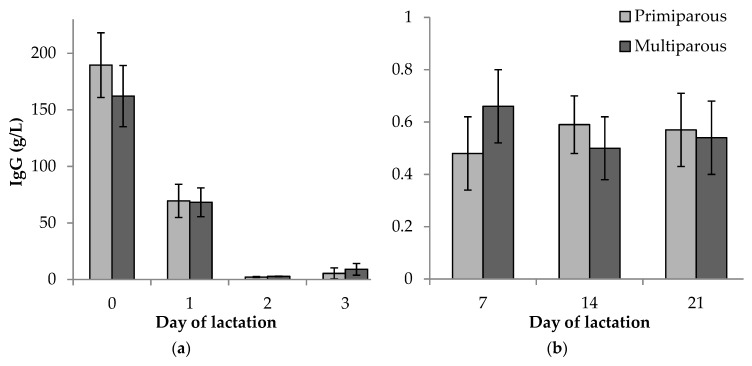
Comparison of total IgG concentration (g/L) between primiparous and multiparous sows in: (**a**) colostrum and transient milk (days 0 to 3); and (**b**) milk in later stages of lactation (days 7 to 21). Error bars represent the standard error of the mean (± SEM).

There was no difference (*p* ≥ 0.05) in concentration of IgG between primiparous and multiparous sow colostrum or milk at any timepoint (Figure 1).

### 3.3. Protein

**Figure 2:**
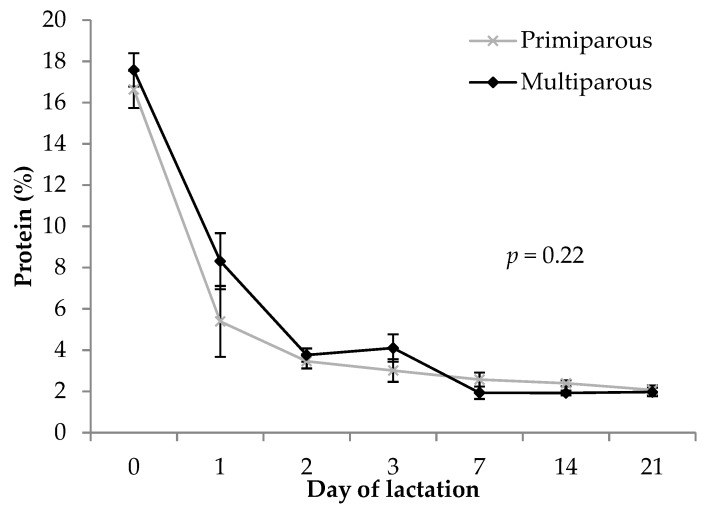
Comparison of total protein concentration (%) in colostrum and milk between primiparous and multiparous sows. The *p-*value stated is the effect of the parity by timepoint interaction. Error bars represent the standard error of the mean (± SEM).

Total protein in colostrum and milk was not different between primiparous and multiparous sows (5.1 ± 0.3 vs. 5.7 ± 0.3%, respectively; *p* = 0.16). Protein decreased over the course of lactation (*p* < 0.001), and this trend was similar in both primiparous and multiparous sow lacteal secretions (interaction *p* = 0.22; Figure 2).

### 3.4. Lactose

**Figure 3:**
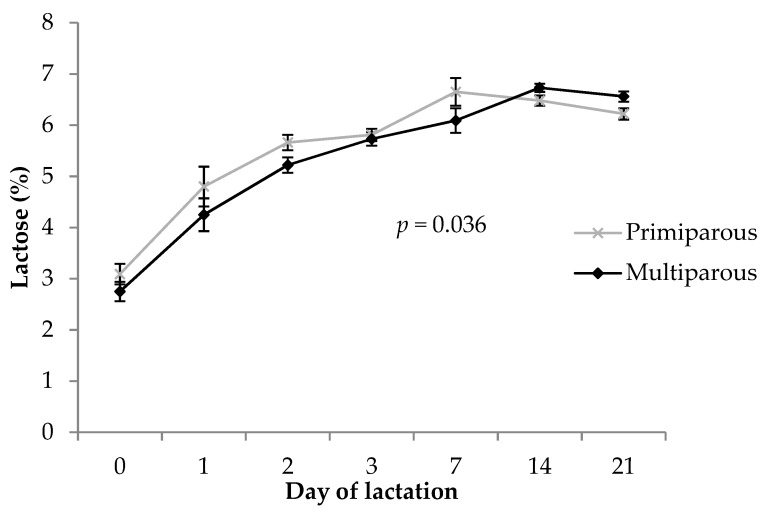
Comparison of total lactose concentration (%) in colostrum and milk between primiparous and multiparous sows. The *p-*value stated is the effect of the parity by timepoint interaction. Error bars represent the standard error of the mean (± SEM).

There was a significant (*p* = 0.036) parity by timepoint interaction effect on lactose content of colostrum and milk, increasing early in lactation for both primiparous and multiparous sows, and the decrease occurring earlier between days 7 and 21 in primiparous sows compared to between days 14 and 21 in multiparous sows (Figure 3). Lactose content was similar (*p* ≥ 0.10) at days 0, 1, 3, and 7 between primiparous and multiparous sows, tended to be higher (*p* = 0.063) at day 2 in primiparous sows, and was higher at days 14 and 21 (*p* = 0.077 and *p* = 0.040, respectively) in multiparous sows. There was no main effect of parity (*p =* 0.21) on colostrum and milk lactose concentration (5.5 ± 0.1% for primiparous sows and 5.3 ± 0.1% for multiparous sows).

### 3.5. Fat

**Figure 4:**
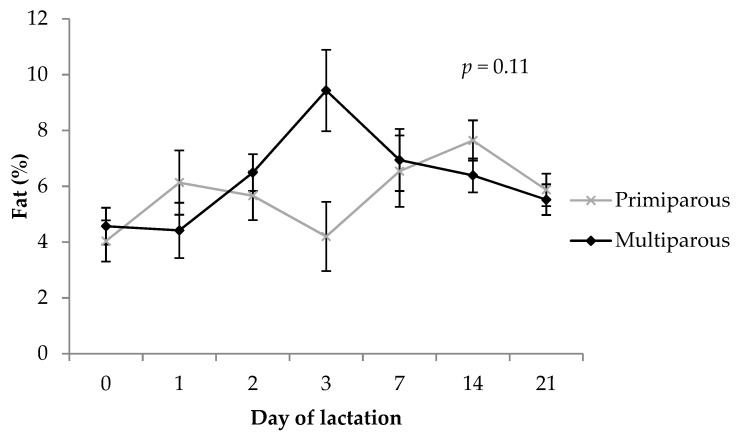
Comparison of total fat concentration (%) in colostrum and milk between primiparous and multiparous sows. The *p-*value stated is the effect of the parity by timepoint interaction. Error bars represent the standard error of the mean (± SEM).

There was no main effect of parity on total fat concentration in colostrum and milk (*p* = 0.34; 5.7 ± 0.4% for primiparous sows vs. 6.3 ± 0.4% for multiparous sows). Overall, fat content increased (*p* = 0.024) from farrowing until day 14 and decrease again by day 21 prior to weaning. Multiparous sow milk increased in total fat content at day 3 whereas this timepoint represented a drop in total fat in primiparous sow milk (Figure 4; parity x timepoint interaction, *p* = 0.11). 

### 3.6. Net Energy Content

**Figure 5:**
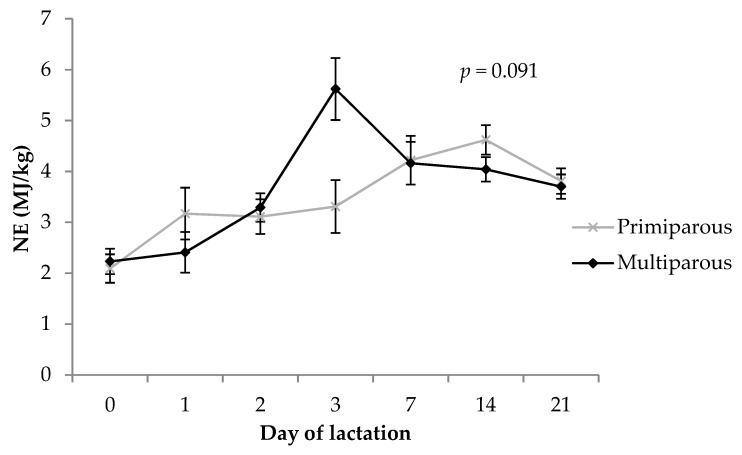
Comparison of calculated NE (MJ/kg) in colostrum and milk between primiparous and multiparous sows. The *p-*value stated is the effect of the parity by timepoint interaction. Error bars represent the standard error of the mean (± SEM).

There was no difference (*p* = 0.46) in NE content of colostrum and milk between primiparous and multiparous sows (3.48 ± 0.16 vs. 3.64 ± 0.15 MJ/kg, respectively). Net energy content changed over time (*p* < 0.001) and followed a similar pattern to total fat (Figure 5). A trend was observed for the parity by timepoint interaction (*p* = 0.091), with similar energy levels in primiparous and multiparous sow milk at all timepoints except day 3, where it was lower (*p* = 0.017) in primiparous sows compared to multiparous sows.

## 4. Discussion

There were minor differences in colostrum and milk composition between primiparous and multiparous sows in the current study. Profiles of IgG, total protein, total fat, and NE were relatively similar between both groups throughout lactation. Differences existed in total lactose in late lactation, where levels seemed to decrease earlier in primiparous sows (around the second week of lactation) compared to older sows (into the third week of lactation). It is, therefore, more likely that the underperformance of gilt progeny compared to sow progeny is attributable to other factors. This is probably a consequence of lower colostrum and milk production in primiparous sows compared to multiparous sows [5,6,30,31], and subsequently a reduction in colostrum and milk available to the piglets, rather than poorer quality of the colostrum and milk itself. It is also possible that there is reduced gastrointestinal permeability in gilt progeny compared to sow progeny [32], limiting their ability to absorb certain components within colostrum and (or) milk. Restriction of fetal growth in late gestation may be responsible for a reduction in development of gastrointestinal tissue and skeletal muscle in neonatal gilt progeny [33,34], and these differences have been shown to persist up to weaning [33,35]. Colostrum production may be affected by number and size of mammary cells [16] and therefore may be compromised in primiparous sows. Overall colostrum yield is difficult to measure accurately [36] and is highly variable between animals [16,37,38]. Regardless, data from a study by Declerck et al. [39] indicate that individual piglet colostrum intake is not different between gilt progeny and sow progeny, with similar total colostrum yields between primiparous and multiparous sows [40]. Lower milk production in primiparous sows in comparison to older sows during established lactation [31] may result from a smaller udder [7] and lower litter sizes reducing suckling pressure and limiting milk production in these animals [31], consequently limiting milk consumption and pre-weaning growth in gilt progeny.

It has been assumed that primiparous sows may produce colostrum and milk with lower levels of immune factors than multiparous sows due to having had less time for exposure to pathogens over their lifetime and a relatively naïve immune system compared to older sows [9]. However, this was not reflected in total colostrum and milk IgG concentrations in the current study, with no difference between primiparous and multiparous sows, in agreement with several recent studies [2,41,42,43]. This contrasts with Cabrera et al. [44], Quesnel [38], Klobasa et al. [45] and Inoue et al. [18], who all found that primiparous sows had significantly lower IgG levels in colostrum and (or) milk than multiparous sows. These discrepancies may be due to responses to nutritional changes, as IgG concentration in colostrum is very sensitive to these [16], as well as genetic advances, and the timing of collection of colostrum samples, particularly the initial sample, as IgG levels in colostrum start to change dramatically 4 to 12 h after the initiation of farrowing [37,46]. Udder section sampled and whether fore- or hind-milk are collected are a further source of variability in colostrum and milk composition [18,19,20,29]. In this regard, the ability to detect statistical differences between primiparous and multiparous sows in colostrum and milk composition may have been limited by sample size (*n* = 20) in the current study. Regardless, the concentration of IgG in colostrum and milk decreased significantly over the course of lactation in the current study, in accordance with others [23,37,47]. Due to lack of previous antigen exposure, specific antibody and (or) IgA concentrations in colostrum and milk may be lower in primiparous sows [45,47], and this requires further investigation. 

Gilt progeny have a lower concentration of serum IgG compared to sow progeny [2,41,48,49], and colostrum intake is relative to the body weight of the piglet, with lighter pigs consuming less colostrum [50]. Acquisition of immunoglobulins such as IgG is important for ensuring survival of piglets, and higher piglet serum IgG concentration [51] and colostrum intake [52] are both correlated with an increased survival chance. At birth, piglets are born devoid of brown adipose tissue for thermoregulation [53] and with little circulating immunoglobulins [52,54]. Reduced absorption of immunoglobulins (“gut closure”) at around 24 to 48 h further prevents immunoglobulin from colostrum passing through to the piglets’ bloodstream [55], hence it is critical that piglets receive colostrum as early as possible. This is particularly important for piglets of lighter birth weight [56,57] and, by inference, gilt progeny [1,11]. Furthermore, colostrum intake and IgG absorption can influence a piglets’ own acquired immunity [58,59]. Total protein in colostrum and milk followed the same pattern as IgG in the current study, which is consistent with the findings of others [23,47]. Our observation that total protein in colostrum and milk was not different regarding parity is in agreement with the results of Declerck et al. [40], Baas et al. [60] and Klobasa et al. [47]. This contrasts with the findings of Beyer et al. [31], who found that primiparous sows had lower milk protein than second to fourth parity sows throughout lactation. On the other hand, Szyndler-Nędza [12] found that primiparous sows had higher milk protein than second and third parity sows on day 14 of lactation.

Values obtained for lactose concentration in colostrum and milk in the current study, and the observation that lactose concentration increased over the course of lactation, are consistent with previous reports [5,47]. Similar to protein levels, our results agree with some studies [40,47,60] and are equivocal to others [12,31] in terms of parity differences in lactose colostrum and milk concentrations. A higher lactose concentration in primiparous sow milk compared to multiparous sow milk at day 2 of lactation as seen in the current study may have implications for gut closure in gilt progeny, as lactose from colostrum has been shown to induce closure [61]. Additionally, specific lactase activity (μmol/min/g of protein) in the small intestine has been shown to be higher in gilt progeny than sow progeny in the first 24 h after birth [33] and may have implications for digestion of lactose during this critical period, which should be further investigated. Primiparous sows had lower lactose levels in milk towards the end of lactation compared to multiparous sows, which may indicate lower late lactation milk yields in these younger sows as a result of being unable to keep up with the demands of maximum milk production. Lactose is the major osmotic component in the mammary gland determining milk volume [62], and hence lower milk lactose levels in primiparous sows may suggest that their ability to produce milk in peak lactation is limited compared to older sows. This is also most likely a consequence of smaller litter sizes in primiparous sows, reflected in one less piglet weaned in the current study (although not statistically significant), as the level of suckling stimulus is important for maintaining lactation and milk production increases with litter size [5,6,63].

In support, several studies have found that milk yield increases with increasing parity [6,30,31,63] and it is possible that primiparous sows, even when feed intake is high, are more likely to partition additional energy into their own growth rather than increasing their milk production [8,64,65]. Primiparous sows are in a more catabolic state in late lactation [66,67] compared to older sows, resulting in prolonged wean to estrus intervals and a reduction in reproductive performance in the second parity [68,69]. Uptake of lactose into the mammary gland and the transfer of lactose to milk may be disrupted in late lactation due to this negative energy balance, with primiparous sows choosing to conserve glucose for metabolism rather than milk production, which could help to explain the drop in lactose concentration. Lactose is often regarded as the most stable nutritional component in colostrum and milk and is therefore difficult to manipulate by factors such as diet [5]. It may be more appropriate, therefore, to focus on management of primiparous sow litters towards late lactation, by feeding additional milk replacer or creep feed, for example.

Fat is an important source of energy for the newborn piglet. Since these animals have limited body fat reserves at birth, there is little for them to mobilize in periods of fasting and therefore intake of fats through the diet is critical [70]. Fat is the most variable component in sow colostrum and milk [5,16,47] and most sensitive to changes in diet, and this is reflected in the outcomes of the current study. There have been mixed results in previous literature and the current study that are reflective of the highly variable nature of colostrum and milk fat concentrations. Several studies have found that colostral fat is highest in primiparous sows and then drops until at least parity 4 [12,16,17,40,71]. However, Peters and Mahan [17] found a quadratic response to parity, with colostral fat increasing again from the fourth parity. Quesnel et al. [16] and Mahan [71] found that fat content of milk was highest in primiparous sows and second parity sows then decreased thereafter, which contrasted with the findings of Beyer et al. [31] who found that primiparous sows had the lowest milk fat concentration and this increased linearly up until parity 4. 

Total NE in milk followed a similar pattern to fat content, which is not surprising given the high energy content of fat in the equation used to calculate this value [72]. The current study found no parity differences in milk NE, and this contrasted with Beyer et al. [31] who reported gross energy of milk to increase from first to fourth parity. The observation of higher NE of milk at day 3 of lactation in multiparous sows was an interesting finding and may be explained by higher feed intake in these animals, and it was unfortunate that feed intake data was not able to be collected in the current study. Primiparous and multiparous sows increase their feed intake substantially over the first week of lactation to keep up with the high nutrient demands of lactation [5]. However, primiparous sows have been shown to have lower feed intake in lactation compared to multiparous sows and they may be limited in gastric capacity [5,10]. This difference in milk NE at the transient milk stage may reflect a delay in adjustment to the commercial “step-up” feeding regime in primiparous sows, whereby feed on offer is slowly increased in the first few days after farrowing, becoming ad libitum after day 3. Additionally, use of oxytocin has been shown to manipulate mammary tight junctions [21]. This prolongs the colostral phase of lactation and may therefore have played a role in the difference in milk NE at day 3 in this study, as the same volume of oxytocin was administered to each animal regardless of body weight. Therefore, oxytocin may have exerted a more pronounced effect on primiparous sows. However, no differences were seen in IgG or protein levels as would be expected if mammary tight junctions had been altered [21].

## 5. Conclusions

The results from this study indicate that colostrum and milk composition throughout lactation does not differ considerably between primiparous sows and third and fourth parity sows in terms of IgG, total protein, fat, or total NE content. However, primiparous sows may not be able to meet the additional demands of a prolonged lactation, supported by a drop in lactose content in the late stages of lactation in primiparous sows compared to multiparous sows in the current study, and this requires further investigation. Consequently, it seems that reduced growth and higher mortality in gilt progeny compared to sow progeny may be more likely due to them having a lower intake of colostrum and milk, and (or) a relative inability to digest and absorb nutritional components from colostrum and milk during the pre-weaning period.

## Figures and Tables

**Table 1:** Number of observations (*n*) for each trait (including all timepoint observations) for each parity group.

Trait	Number of Observations (*n*)	
	Primiparous	Multiparous
Protein (%)	44	53
Lactose (%)	46	53
Fat (%)	44	54
NE (MJ/kg)	43	53
